# Regulation of Feto-Maternal Barrier by Matriptase- and PAR-2-Mediated Signaling Is Required for Placental Morphogenesis and Mouse Embryonic Survival

**DOI:** 10.1371/journal.pgen.1004470

**Published:** 2014-07-31

**Authors:** Roman Szabo, Diane E. Peters, Peter Kosa, Eric Camerer, Thomas H. Bugge

**Affiliations:** 1 Proteases and Tissue Remodeling Section, National Institute of Dental and Craniofacial Research, National Institutes of Health, Bethesda, Maryland, United States of America; 2 Program of Pharmacology and Experimental Therapeutics, Tufts University School of Medicine, Boston, Massachusetts, United States of America; 3 INSERM U970, Paris Cardiovascular Research Centre, Paris, France; 4 Université Paris-Descartes, Paris, France; University of Calgary, Canada

## Abstract

The development of eutherian mammalian embryos is critically dependent on the selective bi-directional transport of molecules across the placenta. Here, we uncover two independent and partially redundant protease signaling pathways that include the membrane-anchored serine proteases, matriptase and prostasin, and the G protein-coupled receptor PAR-2 that mediate the establishment of a functional feto-maternal barrier. Mice with a combined matriptase and PAR-2 deficiency do not survive to term and the survival of matriptase-deficient mice heterozygous for PAR-2 is severely diminished. Embryos with the combined loss of PAR-2 and matriptase or PAR-2 and the matriptase partner protease, prostasin, uniformly die on or before embryonic day 14.5. Despite the extensive co-localization of matriptase, prostasin, and PAR-2 in embryonic epithelia, the overall macroscopic and histological analysis of the double-deficient embryos did not reveal any obvious developmental abnormalities. In agreement with this, the conditional deletion of matriptase from the embryo proper did not affect the prenatal development or survival of PAR-2-deficient mice, indicating that the critical redundant functions of matriptase/prostasin and PAR-2 are limited to extraembryonic tissues. Indeed, placentas of the double-deficient animals showed decreased vascularization, and the ability of placental epithelium to establish a functional feto-maternal barrier was severely diminished. Interestingly, molecular analysis suggested that the barrier defect was associated with a selective deficiency in the expression of the tight junction protein, claudin-1. Our results reveal unexpected complementary roles of matriptase-prostasin- and PAR-2-dependent proteolytic signaling in the establishment of placental epithelial barrier function and overall embryonic survival.

## Author Summary

Development of mammalian embryos is dependent on an efficient exchange of nutrients, oxygen, and waste products between the mother and the embryo. The interface between the two systems is provided by the placenta in a form of a specialized epithelium that both facilitates the transport of molecules between the mother and the embryo and screens the substances that can pass between the maternal and fetal tissues. We now show that two independent signaling pathways that include the serine proteases, matriptase and prostasin, and a G protein-coupled receptor PAR-2, are critical for the establishment of a functional feto-maternal interface by specifically regulating the barrier properties of the placental epithelium. Because aberrant formation of epithelial barriers is an underlying feature of a great variety of human developmental abnormalities, the identification of the two protease-dependent signaling pathways critical for the barrier formation in embryonic tissues may help pinpoint molecular mechanisms involved in the etiology of these conditions.

## Introduction

The development of eutherian mammals requires an efficient exchange of nutrients, oxygen, ions, hormones, and waste products between the maternal and fetal blood. In humans and mice, a functional feto-maternal interface is established in the placenta by the formation of a complex embryonic vascular tree that is submerged in interstitial space filled with maternal blood [1], [2]. Separating the maternal and fetal circulation is a specialized embryo-derived epithelium that functions both to facilitate the transport of molecules between the mother and the embryo, and as a barrier to screen which substances can pass between the maternal and fetal tissues and which cannot. In mice, the epithelium resides in a histologically distinct region of the placenta, termed the labyrinth, and consists of two layers of differentiated polynuclear syncytiotrophoblasts surrounding the fetal vessels and an underlying layer of mononuclear cytotrophoblasts that are in direct contact with the maternal blood [2]. The thickness of the labyrinth, the degree of vascular branching, and the level of expression of transporter proteins within the labyrinth epithelium are the chief determinants of the efficiency of nutrient transfer to the embryo [3].

The ability of the epithelia to restrict free movement of water, solutes, and larger molecules through the interstitial space between the individual epithelial cells is critical for tissue compartmentalization and protection against chemical damage, infection, dehydration, or heat loss [4], [5]. Establishment of paracellular transport barriers across the epithelial layers is primarily achieved by formation of several types of specialized cell-cell junctions that include desmosomes, adherens, and tight junctions [6]. Disruption of the junctional complexes severely compromises epithelial barrier function and is frequently linked to disease in both mice and humans [5], [7]. In the developing embryo, establishment of a functional feto-maternal barrier is critical to protect the fetus from toxins and infection by blood-born pathogens present in maternal circulation during pregnancy, as well as potential ion imbalance, unchecked diffusion of maternal hormones, or an attack by maternal immune system [8]–[12].

Matriptase is a trypsin-like cell surface-associated serine protease that plays an essential role in the homeostasis of a variety of mouse and human epithelia [13]–[15]. Mice with a complete loss of matriptase function die perinatally due to a defect in epidermal barrier function, leading to a fatal dehydration [16], [17]. Likewise, tissue-specific genetic ablation of matriptase revealed a role in epithelial barrier function in a number of other organs, including oral cavity, salivary gland, small intestine, and colon, suggesting a key role of this protease in the establishment of functional epithelial barriers of both single and multi-layered epithelia [18]. Despite a widespread expression in a number of embryonic and extraembryonic epithelia, matriptase, however, is not required for term development in humans and in most mouse strains ([13], [14], [16] and unpublished data). However, the regulation of matriptase activity is essential for the successful completion of the embryonic development, as documented by severe, matriptase-dependent, defects in placental development, neural tube closure, and overall embryonic survival in mice lacking either of the two endogenous serine protease inhibitors, HAI-1 and HAI-2 [19], [20].

Protease-activated receptors (PARs) are a subfamily of the heptahelical G protein-coupled receptors (GPCRs) activated by a proteolytic cleavage within the N-terminal extracellular region, unmasking new amino-terminal residues that then serve as tethered ligands to activate the receptors [21]–[23]. Both in mice and in humans, the subfamily consists of four members, PAR-1 through PAR-4. Mice carrying loss of function mutations in PAR-1 or PAR-2 genes suffer from partial embryonic lethality although born PAR-1- and PAR-2-deficient animals generally show a normal postnatal development and survival [24], [25]. Furthermore, the combined loss of PAR-1 and PAR-2 leads to more than 60% decrease in pre-term and 90% decrease in pre-weaning survival, and to an array of developmental defects that include neural tube defects, edema, and an enlarged pericardium, indicating the requirement of PAR-mediated signaling for the completion of normal embryonic development [26]. Whereas PAR-1, -3, and -4 are activated *in vivo* by the secreted serine protease thrombin and appear to primarily function as part of the blood coagulation cascade, PAR-2 is expressed by a majority of epithelial and endothelial cells and can be activated by a number of soluble and membrane-anchored proteases with trypsin-like activity, but is not efficiently activated by thrombin [23]. Matriptase, in particular, has consistently been shown to efficiently stimulate activation of PAR-2 in a variety of cell-based assays. This, together with a highly overlapping pattern of expression in majority of epithelia, suggested that at least some of the functional effects of matriptase *in vivo* are mediated by PAR-2 and its downstream effectors [26]–[28].

In this study, we report the unexpected observation that matriptase and PAR-2 act during embryogenesis via functionally independent and redundant proteolytic signaling pathways. We show that mouse embryonic development and survival is strongly dependent on the genetic dosage of matriptase and PAR-2 and that the combined loss of both molecules leads to a complete embryonic lethality. Our results reveal unexpected complementary and essential roles of matriptase/prostasin- and PAR-2-dependent signaling in the establishment of placental epithelial tight junctions.

## Results

### Combined loss of matriptase/prostasin- and PAR-2-dependent signaling leads to embryonic lethality

**Figure 1:**
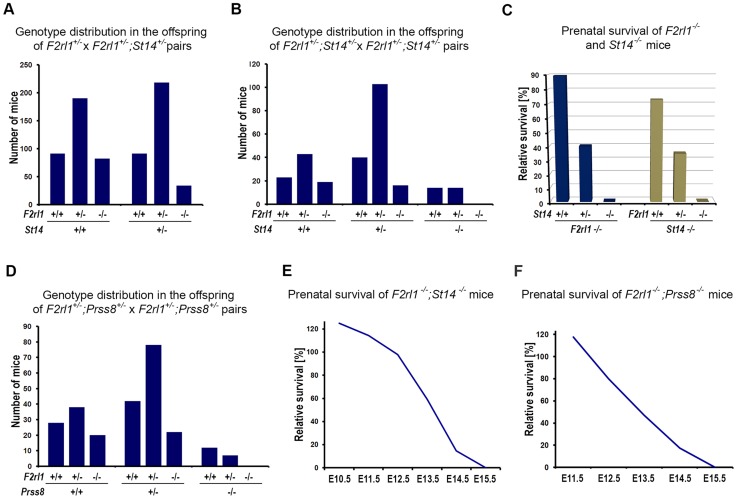
Combined loss of matriptase/prostasin- and PAR-2-dependent proteolytic pathways leads to embryonic lethality. (**A**). Matriptase haploinsufficiency decreases survival of PAR-2-deficient mice. Genotype distribution among 706 pre-weaning offspring from interbred *F2rl1^+/−^*×*F2rl1* ^+/−^ *;St14^+/−^* mice. A normal distribution of *F2rl1* alleles was observed in *St14^+/+^* background, whereas the number of *F2rl1^−/−^* mice heterozygous for matriptase was significantly decreased (P<0.0001). (**B**). Genotype distribution among 272 newborn offspring from interbred *F2rl1^+/−^;St14^+/−^*×*F2rl1* ^+/−^;*St14^+/−^* mice. *F2rl1* alleles were found in the expected Mendelian ratio in *St14^+/+^* mice, whereas numbers of *F2rl1^−/−^;St14^+/−^* and *F2rl1^+/−^;St14^−/−^* mice was significantly reduced, and no *F2rl1^−/−^;St14^−/−^* mice were observed (P<0.0001). (**C**). Relative prenatal survival of PAR-2-deficient (*F2rl1^−/−^*, blue bars) and matriptase-deficient (*St14^−/−^*, green bars) mice shown in (**B**) as a function of gene dosage of *St14* and *F2rl1* genes, respectively. Survival of PAR-2-deficient animals decreased from 90% in *St14^+/+^* background, to 40% in *St14^+/−^* and 0% in *St14^−/−^* background. Similarly, survival of matriptase-deficient mice decreased from 73% in *F2rl1^+/+^* animals to 35% in *F2rl1^+/−^* and 0% in the PAR-2-deficient *F2rl1^−/−^* animals. (**D**). Genotype distribution among 247 newborn offspring from interbred *F2rl1^+/−^;Prss8^+/−^*×*F2rl1* ^+/−^;*Prss8^+/−^* mice. *F2rl1* alleles were found in the expected Mendelian ratio in *Prss8^+/+^* mice, whereas numbers of *F2rl1^−/−^;Prss8^+/−^* and *F2rl1^+/−^;Prss8^−/−^* mice was significantly reduced, and no *F2rl1^−/−^;Prss8^−/−^* mice were observed (P<0.0005). (**E, F**). Survival of *F2rl1^−/−^;St14^−/−^* (**E**) and *F2rl1^−/−^;Prss8^−/−^* (**F**) embryos before birth, relative to the expected Mendelian distribution. (**E**). PAR-2/matriptase double-deficient embryos were detected in expected numbers at or before E12.5, followed by a decrease to 59% at E13.5 (N = 251), and 15% at E14.5 (N = 90). No surviving *F2rl1^−/−^;St14^−/−^* animals were detected at or after E15.5. (**F**). PAR-2 and prostasin double-deficient embryos were detected in expected numbers at E11.5, followed by a gradual decrease to 80% at E12.5 (N = 90), 47% at E13.5 (N = 176), and 17% at E14.5 (N = 68). No surviving *F2rl1^−/−^;Prss8^−/−^* animals were detected at or after E15.5.

Matriptase has consistently been shown to efficiently stimulate activation of PAR-2 in cell-based assays, leading to a conclusion that at least some of its physiological functions may be mediated by PAR-2 [26]–[28]. However, our recent work tentatively suggested that in the context of mouse embryonic development, matriptase and its partner protease prostasin may act independently of PAR-2, based on the findings that: (i) developmental defects observed in mice lacking endogenous matriptase inhibitor, HAI-2, can be rescued by matriptase or prostasin deficiency but not by PAR-2 deficiency, and (ii) a genetic inactivation of matriptase fails to reproduce phenotypes associated with PAR-2 deficiency in mice lacking PAR-1 [29]. The HAI-2; PAR-2 double-deficient mice (*Spint2^−/−^;F2rl1^−/−^*) used in that study were mostly generated by interbreeding of HAI-2; PAR-2 double-heterozygous and HAI-2; PAR-2; matriptase triple-heterozygous mice (*Spint2^+/−^;F2rl1^+/−^*×*Spint2^+/−^;F2rl1^+/−^;St14^+/−^*). Unexpectedly, a detailed review of the genotype distribution among the offspring from these breeding pairs at weaning showed that the survival of PAR-2-deficient mice was strongly dependent on the number of active alleles of matriptase. Thus, in offspring carrying two active alleles of matriptase (*St14^+/+^*, matriptase wildtype) about 90% of the *F2rl1^−/−^* mice survived until weaning (Figure 1A and Table S2), consistent with the previously reported 10–30% pre-term lethality among the *F2rl1^−/−^* mice [25], [26]. However, in the offspring that lacked one functional allele of matriptase (*St14^+/−^*, matriptase heterozygous), the survival of the *F2rl1^−/−^* mice decreased to less than 40% compared to the expected Mendelian distribution (Figure 1A and Table S2). To further analyze this dramatic loss of survival and to test the survival of PAR-2-deficient mice in the complete absence of matriptase, we established a new cohort of breeders double-heterozygous for both matriptase and PAR-2 (*F2rl1^+/−^;St14^+/−^*). Inspection of the allele distribution in newborn offspring (Figure 1B) confirmed the highly significant increase in embryonic lethality (Figure 1C) among *F2rl1^−/−^* animals carrying one active allele of matriptase (*F2rl1^−/−^;St14^+/−^*, 40% pre-natal survival), compared to matriptase wildtype animals (*F2rl1^−/−^;St14^+/+^*, 90% pre-natal survival). Furthermore, analysis of the 272 newborn mice from the *F2rl1^+/−^;St14^+/−^* breeding pairs did not identify any *F2rl1^−/−^;St14^−/−^* pups, indicating a complete pre-term lethality of *F2rl1^−/−^* mice in the absence of matriptase (Figure 1B and 1C, Table S3). Similarly, embryonic survival of matriptase-deficient mice was strongly dependent on the gene dosage of the PAR-2 gene, as indicated by73%, 35%, and 0% pre-term survival of *St14^−/−^* mice carrying two, one, or no active alleles of the PAR-2 gene, respectively (Figure 1B and C, Table S3).

Activation of matriptase during embryonic development was recently shown to be dependent on the activity of the GPI-anchored serine protease prostasin (PRSS8/CAP1), indicating that the two proteases are part of the same proteolytic signaling pathway [29]. Consistent with this finding, analysis of the 247 newborn offspring from the *Prss8^+/−^;F2rl1^+/−^* breeding pairs also showed a diminished survival of mice with low combined dosage of *Prss8* and *F2rl1* genes. Specifically, the pre-term survival of PAR-2-deficient mice decreased from 93% in prostasin wildtype animals (*Prss8^+/+^;F2rl1^−/−^*) to 62% and 0% in mice carrying one (*Prss8^+/−^;F2rl1^−/−^*) or no (*Prss8^−/−^;F2rl1^−/−^*) active alleles of prostasin (Figure 1D, Table S4). These data show that the pre-term survival of mice is completely dependent on either PAR-2- or matriptase/prostasin-mediated proteolytic signaling.

### Matriptase; PAR-2 double-deficient mice die at midgestation despite normal development

We next investigated the offspring of interbred *F2rl1^+/−^*;*St14^+/−^*×*F2rl1^+/−^*;*St14^+/−^* and *F2rl1^−/−^*;*St14^+/−^*×*F2rl1^+/−^*;*St14^+/−^* mice at various stages of embryonic development to further characterize the lack of term survival in mice double-deficient for PAR-2 and matriptase. Analysis of embryos extracted between E10.5–E12.5 revealed normal distribution of *F2rl1* and *St14* alleles, and nearly all of the *F2rl1^−/−^*; *St14^−/−^* embryos extracted before E12.5 were alive and appeared normal, indicating that the pre-implantation and the early post-implantation development and survival were not affected by the combined loss of *F2rl1* and *St14* gene function (Figure 1E). After E12.5, however, the survival of the *F2rl1^−/−^*;*St14^−/−^* animals dramatically decreased to 59% at E13.5, 14% at E14.5, and no living *F2rl1^−/−^*;*St14^−/−^* embryos were identified at or after E15.5 (Figure 1E and Table S5). Similarly, analysis of the offspring from *F2rl^+/−^*;*Prss8^+/−^* breeding pairs found an expected number of living *Prss8^−/−^*;*St14^−/−^* embryos at or before embryonic day 12.5, followed by a dramatic decrease in survival to 47% at E13.5, 17% at E14.5, and no surviving double-deficient embryos identified at and after E15.5 (Figure 1F and Table S6).

**Figure 2:**
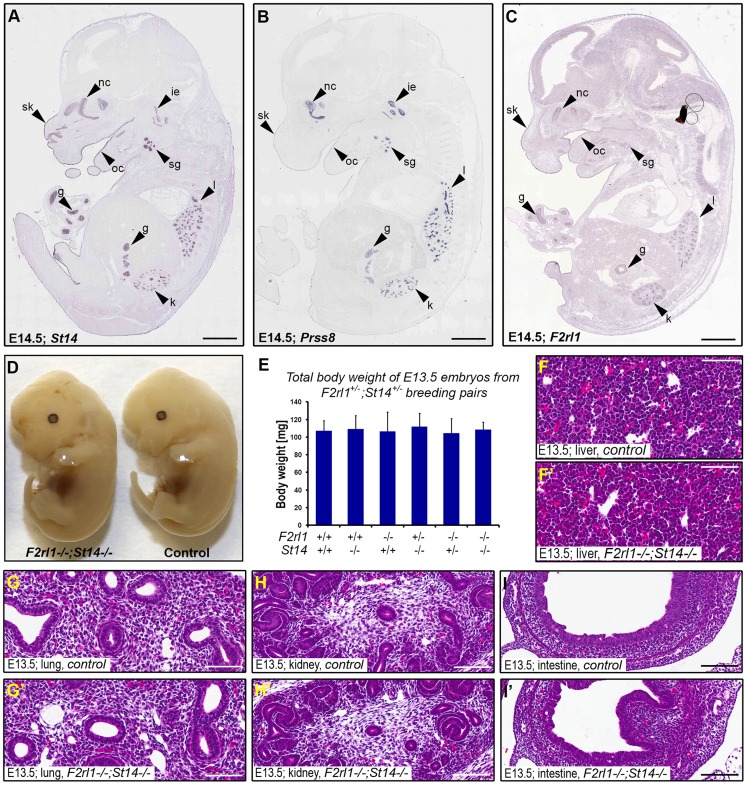
Loss of PAR-2 and matriptase does not affect development of the embryo proper. (**A–C**). Evaluation of the expression pattern of *St14* (**A**), *Prss8* (**B**), and *F2rl1* (**C**) genes in E14.5 mouse embryo by *in situ* hybridization obtained from the Eurexpress digital transcriptome atlas. All three genes show highly overlapping pattern of expression in the epithelia of developing skin (sk), oral (oc) and nasal (nc) cavities, salivary gland (sg), lungs (l), kidney (k), and gut (g). Matriptase and prostasin, but not PAR-2, were also detected in the developing structures of the inner ear (ie). (**D**). Macroscopic appearance of *F2rl1^−/−^;St14^−/−^*, and wild-type littermate control embryos at E13.5. No obvious developmental abnormality was observed in the PAR-2 and matriptase double-deficient animals. (**E**) Total body weight of E13.5 offspring from *F2rl1^+/−^;St14^+/−^* breeding pairs. None of the embryos with a decreased combined gene dosage of *F2rl1* and *St14* genes showed any signs of growth retardation. (**G–I′**). H&E staining of embryonic tissues with the highest relative expression of PAR-2 and matriptase. A comparative histological analysis of liver (**F, F′**), lungs (**G, G′**), kidneys (**H, H′**), and intestines (**I, I′**) from the control (**F, G**, **H**, and **I**) and littermate *F2rl1^−/−^;St14^−/−^* (**F′, G′**, **H′**, and **I′**) E13.5 embryos indicates normal development of embryonic tissues in the absence of PAR-2 and matriptase function. Scale bars: (**A–C**) 1 mm, (**F–I′**) 100 um.

Matriptase, prostasin, and PAR-2 all are membrane-anchored proteins and are believed to function primarily at the surface of the expressing cells. To help us identify embryonic structures potentially affected by the combined loss of matriptase/prostasin- and PAR-2-dependent activities, we looked for cells simultaneously expressing *St14*, *Prss8*, and *F2rl1* in mid-gestation embryos using the publicly available Eurexpress transcriptome atlas database [30]. All three genes were detected in a number of embryonic tissues at E14.5, showing an extensive co-expression in developing epithelia of skin, oral and nasal cavities, salivary gland, lungs, kidneys, and gastrointestinal tract (Figure 2A–C). Surprisingly, despite the widespread co-expression, a detailed inspection of the living E12.5–14.5 *F2rl1^−/−^;St14^−/−^* or *F2rl1^−/−^*;*Prss8^−/−^* embryos failed to identify any obvious developmental abnormality. The double-deficient mice did not develop any signs of internal bleeding, edema, or enlarged pericardium typical for many mouse strains exhibiting mid-gestational lethality (Figure 2D), and did not differ in size or total body weight from their wild-type littermate controls (Figure 2D and 2E). Furthermore, histological analysis of living E11.5–14.5 *F2rl1^−/−^;St14^−/−^* embryos did not reveal any obvious defects in the development of any of the epithelia with detectable levels of matriptase, prostasin, and PAR-2 gene expression (see above) or any other major organs, which are typically affected in mouse strains that exhibit mid-gestational lethality, including liver [31] (Figure 2F–I′).

### Combined loss of matriptase and PAR-2 leads to defects in development of the placental labyrinth

**Figure 3:**
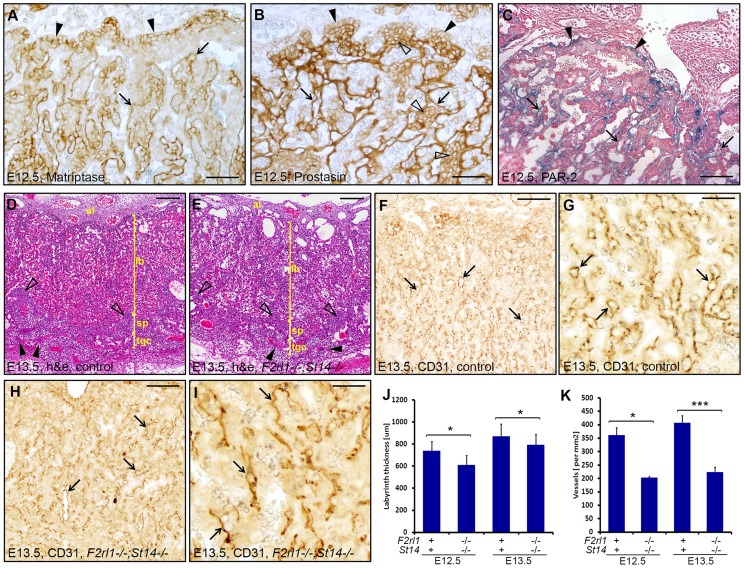
A combined loss of PAR-2 and matriptase leads to underdevelopment of placental labyrinth. (**A, B**). Immunohistochemical detection of matriptase (**A**) and prostasin (**B**) in mouse placenta at E12.5. Expression of both proteins was observed in chorionic epithelium (**A** and **B**, arrowheads) and in the differentiated syncytiothrophoblast layer of the labyrinth (**A** and **B**, arrows). Prostasin, but not matriptase, was also detected in the mononuclear cytotrophoblast cells within the labyrinth (**B**, open arrowheads). (**C**). Histological analysis of PAR-2 expression in the developing placenta from E12.5 *F2rl1-βgal* knock-in embryos. Beta-galactosidase reporter activity was detected both in chorionic epithelium (arrowheads) and in the syncytiothrophoblast layer surrounding fetal vessels within the labyrinth (arrows). (**D, E**). Histological evaluation of the placental development in control (**D**) and *F2rl1^−/−^;St14^−/−^* (**E**) animals at E13.5. H&E staining confirmed the presence of all major structural components of the mouse placenta, including allantoic mesenchyme (al), placental labyrinth (lb), spongiotrophoblast layer (sp, examples with open arrowheads), and trophoblast giant cells (tgc, examples with arrowheads), in the double-deficient embryos (**F–I**). Immunohistochemical visualization of fetal vasculature in the E13.5 placentas. Low (**F, H**) and high (**G, I**) magnification images of the staining for the endothelial cell marker PECAM-1/CD31 in the placentas of the control (**F, G**) and *F2rl1^−/−^;St14^−/−^* (**H,I**) mice shows presence of the branched fetal vascular tree (**F–I**, arrows) within the placental labyrinth of the PAR-2/matriptase double-deficient animals, although the apparent vascular density is lower than in the wildtype littermate control tissues. (**J, K**). Quantification of the thickness of the placental labyrinth (**J**) and the number of fetal vessels (**K**) within the labyrinth of control (*F2rl1^+^;St14^+^*) and *F2rl1^−/−^;St14^−/−^* E12.5 and E13.5 placentas. The measurements show decreased thickness and vascularization of the labyrinth layer in *F2rl1^−/−^;St14^−/−^* placentas at E12.5 and E13.5. P values: *<0.05, **<0.01, ***<0.001, Student's t-test, two-tailed. Scale bars: (**A, B**) 50 µm, (**C**) 100 um, (**D, E, F, H**) 200 µm, (**G, I**) 50 µm.

Failure to identify any specific defect associated with a combined loss of matriptase and PAR-2 in the embryo proper prompted us to examine development of other tissues of fetal origin. Consistent with our previous findings, immunohistochemical analysis revealed strong expression of matriptase and prostasin in the epithelial compartment of the placental labyrinth at midgestation (Figure 3A and 3B) [19], [20], [29]. To address the expression of PAR-2 in the absence of suitable antibodies, we employed a previously generated knock-in mouse strain that carries a β-galactosidase reporter construct under the control of the endogenous promoter of *F2rl1* gene [32]. Analysis of the distribution of β-galactosidase activity in mouse placental tissues at E12.5 revealed that, similar to matriptase and prostasin, expression of the *F2rl1* gene is found predominantly in the epithelium of the chorion and in the syncytiotrophoblast layer lining fetal endothelium within the labyrinth (Figure 3C). These data identify labyrinthine epithelium as the placental population most likely to be affected by a combined loss of matriptase/prostasin and PAR-2.

Initial histological evaluation indicated that all embryo-derived placental structures, including layers of trophoblast giant cells, spongiotrophoblasts, placental labyrinth, and allantoic mesenchyme were all formed in the placentas of *F2rl1^−/−^;St14^−/−^* embryos and there were no signs of placental edema (Figure 3D and 3E). Furthermore, immunohistochemical staining for the endothelial cell marker CD31/PECAM1 demonstrated the presence of a highly branched fetal vasculature in close proximity to maternal blood lacunae within the labyrinth of *F2rl1^−/−^;St14^−/−^* placentas (Figure 3F–3I), indicating that the combined loss of PAR-2 and matriptase does not block any of the major morphogenetic processes involved in placental development. However, a more detailed morphometric analysis revealed noticeable quantitative changes in the development of the labyrinth structure. The overall thickness of the labyrinth, defined as the maximum perpendicular distance between the undifferentiated chorionic epithelium and the labyrinth supporting spongiotrophoblast layer, was reduced by 10–15% in placentas from *F2rl1^−/−^;St14^−/−^* embryos at E12.5 and E13.5 (P<0.05, Figure 3J). This was also reflected in a 15% reduction of total volume of the labyrinth in *F2rl1^−/−^;St14^−/−^* placentas at E13.5, as determined by the Cavalieri stereological technique (Figure S1). More importantly, the complexity of the fetal-derived vasculature within the labyrinth that mediates bidirectional transport of molecules between the mother and the embryo was substantially reduced, as indicated by more than 40% decrease in the number of CD31/PECAM1-positive fetal capillary profiles found within the labyrinth area of double-deficient placentas at E12.5 and E13.5 (Figure 3F–3I and Figure 3K, P<0.01 and 0.001, respectively, Student's t-test, two-tailed). These data indicate that the combined loss of matriptase-prostasin- and PAR-2-dependent signaling interferes with the development of the feto-maternal interface.

### Placental expression of matriptase rescues embryonic survival of matriptase/PAR-2 double deficient mice

**Figure 4:**
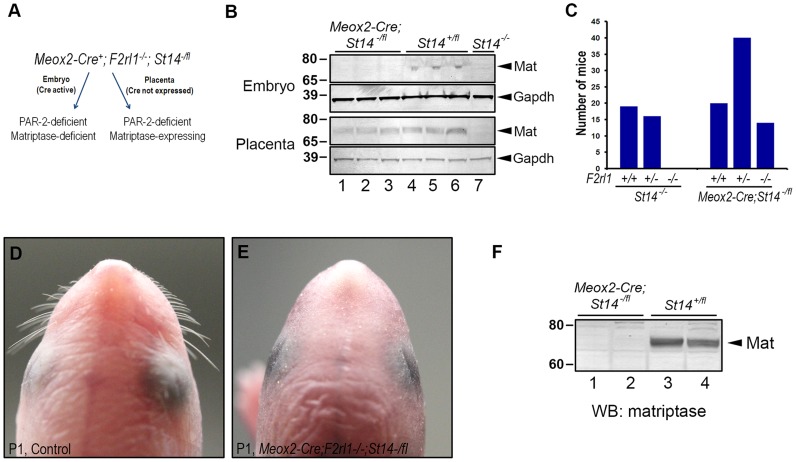
Placental expression of matriptase restores survival in PAR-2 and matriptase double-deficient embryos. (**A**). Schematic depiction of the strategy used to assess contribution of placental matriptase to the survival of the *F2rl1^−/−^;St14^−/−^* embryos. *Meox2-Cre* allele that drives the expression of Cre recombinase to the embryonic but not placental tissues was introduced into the mice carrying one null and one conditional allele of matriptase gene (*St14^−/fl^*). The resulting mice retain expression of matriptase in the placenta but not in any of the embryonic tissues. Both the embryo and the placenta are also PAR-2-deficient (*F2rl1^−/−^*). (**B**). Western blot analysis of matriptase expression in the embryo (top) and the placenta (bottom) of the E13.5 *Meox2-Cre;St14^−/fl^* (lanes 1–3), and their *St14^+/fl^* (lanes 4–6) and *St14^−/−^* (lane 7) littermate control animals. Both the embryos and the placentas of the control mice showed detectable levels of matriptase protein (arrowheads on the right), whereas only placental tissue retained matriptase expression in the *Meox2-Cre;St14^−/fl^* embryos. No matriptase expression was detected in either embryos or placentas of animals carrying two knockout alleles of matriptase (*St14^−/−^*). GAPDH signal is shown to indicate equal loading. Positions of molecular weight markers (kDa) are shown on left. (**C**). Allele distribution of the *F2rl1* gene among newborn matriptase-deficient offspring from interbred *F2rl1^+/−^;St14^+/−^*×*F2rl1* ^+/−^;*St14^+/−^* (*St14^−/−^*, left panels) or *Meox2-Cre;F2rl1^+/−^;St14^+/−^*×*F2rl1^+/−^;St14^−/fl^* (*Meox2-Cre;St14^−/fl^*, right panels) breeder mice. Placental expression of matriptase restores embryonic survival of the PAR-2 and matriptase double-deficient mice. (**D, E**). Macroscopic appearance of the head of control (**D**) or *Meox2-Cre;F2rl1^−/−^;St14^−/fl^* (**E**) mice at birth. The *Meox2-Cre;F2rl1^−/−^;St14^−/fl^* newborns reproduce phenotypes of mice with complete matriptase deficiency, including the lack of whiskers. (**F**). Western blot analysis of matriptase expression in two *Meox2-Cre;F2rl1^−/−^;St14^−/fl^* (lanes 1–2) and two littermate control (lanes 3–4) newborn mice. No residual expression of matriptase protein (arrowhead on the right) was detected in the *Meox2-Cre;F2rl1^−/−^;St14^−/fl^* mice. Positions of molecular weight markers (kDa) are shown on left.

To test the possibility that the loss of viability of *F2rl1^−/−^;St14^−/−^* mice at midgestation results from a defect in the development of placental rather than embryonic tissues, we next investigated survival of PAR-2-deficient mice with a specific inactivation of matriptase in the embryo proper. To that end, PAR-2 heterozygous breeders carrying conditional *St14* allele (*F2rl1^+/−^;St14^fl/fl^*) were crossed to *F2rl1^+/−^;St14^+/−^* animals expressing Cre recombinase under the control of the endogenous *Meox2* promoter. *Meox2* expression is ubiquitous in mouse embryonic tissues as early as at E6.5, but is missing from any of the extraembryonic structures, including the placenta [33]. As a result, whereas both the embryos and the placentas of the *Meox2-Cre^+^;F2rl1^−/−^;St14^−/fl^* offspring would be devoid of PAR-2 expression, only the embryos will also be rendered matriptase-deficient due to the recombination of the remaining floxed *St14* allele (Figure 4A). A potential survival of *Meox2-Cre^+^;F2rl1^−/−^;St14^−/fl^* embryos would therefore demonstrate a critical contribution of placental matriptase to the embryonic survival of PAR-2 and matriptase double-deficient mice, whereas lack of survival would be an indication that embryonically-expressed matriptase determines viability of these animals.

Western blot analysis of protein lysates from the E13.5 embryos and placentas showed a complete loss of expression of matriptase protein in the *Meox2-Cre^+^;F2rl1^−/−^;St14^−/fl^* mid-gestation embryos, whereas the expression of the protease in the corresponding placentas was easily detectable (Figure 4B), thus confirming the efficient, embryo-specific inactivation of the *St14* conditional allele. However, despite being matriptase- and PAR-2-deficient, *Meox2-Cre^+^;F2rl1^−/−^;St14^−/fl^* embryos survived to term and were detected among the newborn offspring from the *F2rl1^+/−^;St14^fl/fl^*×*Meox2-Cre^+^;F2rl1^+/−^;St14^+/−^* breeding pairs in the expected ratio (Figure 4C). These mice recapitulated all of the phenotypes previously observed in matriptase knockout mice, including dry skin, and lack of whiskers, and showed no residual expression of matriptase in newborn tissues (Figure 4D–4F). These findings document that the expression of matriptase in the embryo proper is dispensable for the pre-term development and survival of PAR-2; matriptase double-deficient mice.

### Matriptase and PAR-2 promote placental barrier function

**Figure 5:**
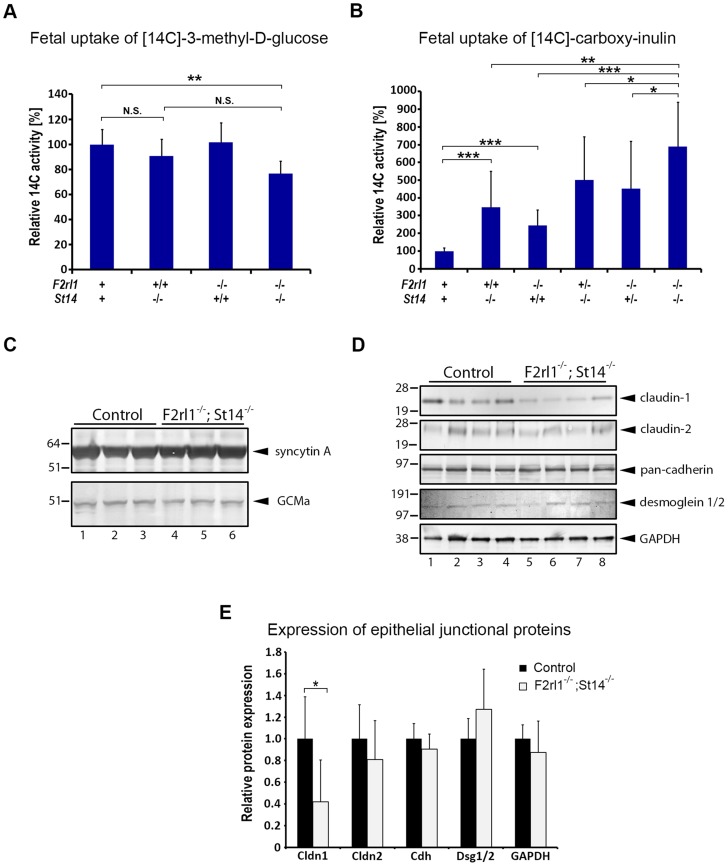
Loss of matriptase and PAR-2 function impairs formation of the feto-maternal barrier. (**A**). Relative uptake of the transcellular transport marker 3-methyl-D-glucose injected into the maternal bloodstream by E12.5–13.5 embryos. Placentas of matriptase (*F2rl1^+/+^,St14^−/−^*) or PAR-2 (*F2rl1^−/−^;St14^+/+^*) single-deficient embryos supported glucose transport at a rate comparable to the wildtype littermate controls (*F2rl1^+^;St14^+^*), whereas matriptase and PAR-2 double-deficient mice (*F2rl1^−/−^;St14^−/−^*) showed about 20% decrease in the glucose uptake (P<0.01). (**B**). Relative uptake of the paracellular transport marker carboxy-inulin injected into the maternal bloodstream by E12.5–13.5 embryos. Diffusion of carboxy-inulin across the placental epithelium was strongly dependent on gene dosage of both matriptase and PAR-2, showing 247% and 145%, respectively, increase in *St14^−/−^* and *F2rl1^−/−^* single-deficient embryos, and 402% and 351% increase, respectively, in *F2rl1^+/−^;St14^−/−^* and *F2rl1^−/−^;St14^+/−^* embryos, compared to the wildtype littermate controls. Highest levels of inulin transfer across the placenta, 589% above the control, was observed in mice with a combined matriptase and PAR-2 deficiencies (*F2rl1^−/−^;St14^−/−^*). (**C**). Expression of placental labyrinth differentiation markers syncytin A (upper panel) and GCMa (lower panel) in the placental tissues from E12.5 matriptase- and PAR-2-expressing (control, lanes 1–3) or matriptase- and PAR-2 double-deficient (*F2rl1^−/−^;St14^−/−^*, lanes 4–6) embryos. No change in the expression of either of the two proteins was detected. (**D, E**). Western blot analysis (**D**) and protein signal quantification (**E**) of the major structural components of epithelial tight junctions (claudin-1 and -2), adherens junctions (pan-cadherin), and desmosomes (desmoglein-1 and -2), or GAPDH as the protein loading control in the placental tissues from E12.5 matriptase- and PAR-2-expressing (control, lanes 1–4) or matriptase and PAR-2 double-deficient (*F2rl1^−/−^;St14^−/−^*, lanes 5–8) embryos. No significant differences were observed in the expression of claudin-2, cadherins, or desmoglein 1 and 2, whereas the expression of claudin-1 was significantly reduced. P values: *<0.05, ** <0.01, *** <0.001, N.S. = not significant, Student's t-test, two-tailed.

The labyrinthine epithelium in mice is a principal component of the feto-maternal barrier, serving both to transport and to screen the substances passing between the maternal blood and the fetal tissues. To test whether the loss of matriptase and/or PAR-2 expression interferes with either of these functions, we next investigated the rate of molecular transport across the placental epithelium. Many nutrients, including glucose, are carried across the placenta by facilitated diffusion using an array of cell-surface transporters expressed by placental epithelial cells [34]. The efficiency of glucose uptake by the embryo can therefore be used as a quantitative measure of placental nutrient transport capacity [35]. To that end, radioactively-labeled 3-O-[methyl-^14^C]-D-glucose was injected into the bloodstream of pregnant females at E12.5 or E13.5, followed by embryo extraction after 2 minutes and measurement of fetal uptake of the [^14^C] label. Consistent with the notable underdevelopment of placental labyrinth layer in matriptase/PAR-2 double-deficient animals (see above), uptake of glucose by the *F2rl1^−/−^;St14^−/−^* embryos decreased by about 20% compared to wild-type littermate controls (P<0.05, Figure 5A). However, this relatively modest reduction in the transport rate together with a lack of any of the typical indications of embryo malnutrition, such as reduced size, gross underdevelopment, or enlarged pericardium, did not conclusively demonstrate an insufficient placental transport as the primary cause of embryonic lethality in the double knockouts.

In addition to the transport of gases and nutrients, placenta also fulfills a critical function of a barrier protecting the fetus against unchecked diffusion of substances, including ions, hormones, components of maternal immune system, or blood-born pathogens from maternal to fetal tissues. To test the ability of the placental epithelium to act as a barrier against passive diffusion, pregnant females were injected at E12.5 or 13.5 with the radioactively-labeled polysaccharide inulin, which can cross epithelial layers solely by a paracellular route due to lack of specific transporters [35], [36]. Interestingly, measurement of uptake of [^14^C] label revealed dramatic differences in inulin diffusion into the embryos of different genotypes. Thus, genetic inactivation of either matriptase or PAR-2 alone resulted in 247% and 145% increase in placental permeability (Figure 5B). This defect was further exacerbated in matriptase-deficient animals heterozygous for PAR-2 (*F2rl1^+/−^;St14^−/−^*, 396% increase) or PAR-2-deficient animals heterozygous for matriptase (*F2rl1^−/−^;St14^+/−^*, 351% increase) (Figure 5B). Finally, the highest level of inulin diffusion was observed in double-deficient embryos (*F2rl1^−/−^;St14^−/−^*, 589% increase, Figure 5B). These data indicate a dramatic loss of feto-maternal barrier function in mice with decreased levels of matriptase and/or PAR-2, the extent of which precisely correlates with the overall embryonic survival in these animals.

### Combined loss of matriptase and PAR-2 leads to a selective decline in claudin-1 expression

Loss of placental barrier can, in principle, be a result of either: (i) an indirect effect due to an impairment of terminal differentiation and subsequent barrier acquisition within the labyrinthine epithelium, or (ii) a direct alteration of the properties of the barrier-forming epithelial cell-cell junctions in the terminally differentiated trophoblasts. To distinguish between the two possibilities we first analyzed the expression of two of the principal regulators of labyrinth differentiation, transcription factor Glial Cell Missing (GCM) a and syncytin, a fusogenic retroviral protein that mediates terminal differentiation of placental cytotrophoblasts into multinucleated syncytium [37], [38]. Analysis of placental tissues from E12.5 matriptase/PAR-2 double-deficient animals and their wild-type littermate controls did not reveal any obvious changes in the expression level of either of the two markers (Figure 5C), indicating that the process of labyrinth differentiation is not compromised by a combined loss of the two proteolytic pathways. To assess the formation of barrier-promoting epithelial cell-cell junctions, we next analyzed the levels of claudin-1 and -2, cadherins, and desmogleins 1 and 2, as the major structural components of tight junctions, adherens junctions, and desmosomes, respectively. Whereas the expression of cadherins and desmogleins was not affected by the combined loss of matriptase and PAR-2, the expression of tight junction marker claudin-1 but not claudin-2 was severely diminished (Figure 5D and 5E). Matriptase or PAR-2 single-deficient placentas did not exhibit significant changes in claudin-1 expression (Figure S2). These findings suggest that a combined activity of matriptase/prostasin and PAR-2 may regulate permeability of placental labyrinth by specifically altering properties of epithelial tight junctions.

## Discussion

Inadequate placental development may lead not only to miscarriage, but also to poor health and increased risk of chronic disease in born individuals [39]. The current study for the first time reveals that the matriptase-prostasin system plays an important role in placental morphogenesis. Thus, mice with placental ablation of matriptase displayed impaired placental barrier formation, which was synergistically exacerbated by haploinsufficiency for or by complete deficiency of PAR-2. A role for the matriptase-prostasin system in placental morphogenesis would be anticipated because of the temporally and spatially coordinated expression of both membrane-anchored serine proteases and their cognate inhibitors, HAI-1 and HAI-2, in the developing placenta [19], [20], [29], [40] and the need for strict posttranslational regulation of both proteases for normal placentation to occur [19], [20], [29]. However, both matriptase-deficient humans and mice have been reported to complete embryonic development [13], [14], [16], and the role of this proteolytic cascade in development therefore has remained unclear. Because of the coordinated expression of matriptase, prostasin, HAI-1 and HAI-2 in several other developing epithelia, it now appears likely that matriptase/prostasin-initiated cellular signaling may also contribute to the development of several other organ systems in the embryo proper, acting in a redundant manner with other proteolytic systems that provide developmental backups.

Numerous studies have demonstrated a role of PAR-2 signaling, triggered by proteases of the coagulation cascade and by other trypsin-like serine proteases, in promoting both epithelial and endothelial barrier leakage by increasing paracellular permeability [41]–[45]. Our study is the first to identify a context in which PAR-2 signaling promotes epithelial barrier formation, rather than barrier leakage, thus adding another property to this multifunctional receptor. The observed specific deficiency in the expression of tight junction protein claudin-1 could suggest that the two proteolytic pathways directly promote barrier formation of terminally differentiated placental epithelium rather than doing it indirectly by providing morphogenetic signaling enabling the terminal differentiation of placental epithelium and the subsequent paracellular barrier acquisition between terminally differentiated epithelial cells. However, it should be noted that it is not yet clear whether the demise of the matriptase/PAR-2 double-deficient animals can be attributed to the observed defect in the expression of claudin-1, especially considering that claudin-1-deficient mice can complete embryonic development, at least in some mouse genetic backgrounds [46]. A previous report by Buzza and coworkers [47] suggested that matriptase regulates intestinal barrier function in a claudin-2-dependent manner. Our current analysis did not reveal any differences in claudin-2 expression in matriptase/PAR-2 double-deficient placental tissues, suggesting that different mechanisms of proteolysis-mediated regulation of tight junction function may be employed by different tissues and/or at different stages of mouse development.

Previous studies have shown that the matriptase-prostasin system is a potent activator of PAR-2 in a variety of cell-based assays [26]–[28], and PAR-2, matriptase, prostasin, HAI-1 and HAI-2 all are co-expressed in multiple developing and adult epithelia. Our genetic demonstration that the matriptase-prostasin system and PAR-2 are components of two independent and functionally redundant proteolytic pathways during placental morphogenesis therefore is unexpected and raises two unanswered questions: First, what is the identity of the placental protease(s) that activates PAR-2 in the absence of matriptase or prostasin? Second, what is the identity of the non-PAR-2 substrate(s) that is cleaved by the matriptase-prostasin cascade to promote placental morphogenesis? Regarding the former, PARs are activated by members of the S1 family of trypsin-like serine proteases, but not by other serine proteases or by other protease classes [48]–[50]. Previous transcript analysis of developing mouse embryos (in the context of neural tube closure) revealed the expression of a surprisingly large number of secreted and membrane-anchored serine proteases [20], [26]. The identification of the protease(s) that activates PAR-2 during placental morphogenesis by using a candidate epistasis analysis approach therefore may be a challenging undertaking. Regarding the latter question, the matriptase-prostasin cascade has been proposed to execute the cleavage of a wide variety of substrates, including other proteases (urokinase plasminogen activator, kallikrein-5 and -7, stromelysin-1), growth factors (hepatocyte growth factor, platelet-derived growth factor, macrophage-stimulating protein) tyrosine kinases (angiopoietin receptor, subtractive immunization M(+)HEp3 associated 135 kDa protein/CUB domain-containing protein-1, epidermal growth factor receptor) and more [15], [28], [51]–[62], reviewed in [18]. Identification of the specific proteolytic targets for the matriptase-prostasin cascade in the placenta therefore may prove difficult.

In summary, our study is the first to demonstrate a function of matriptase/prostasin- and PAR-2-dependent protease signaling in placental morphogenesis and to show that the two pathways act in an independent and functionally redundant manner to promote formation of the placental barrier.

## Materials and Methods

### Mouse strains

All experiments were performed in an Association for Assessment and Accreditation of Laboratory Animal Care International-accredited vivarium following Standard Operating Procedures. The studies were approved by the NIDCR Institutional Animal Care and Use Committee. All studies were littermate controlled. Matriptase-deficient (*St14^−/−^*), PAR-2-deficient (*F2rl1^−/−^*), and *Meox2-Cre* transgenic mice have been described previously [16], [18], [33], [63]. Prostasin-deficient (*Prss8^−/−^*) mice were generated by standard blastocyst injection of C57BL/6J-derived embryonic stem cells carrying a gene trap insertion in the *Prss8* gene (clone IST10122F12, Texas A&M Institute for Genomic Research, College Station, TX). Details on mouse generation will be published separately. Ear or tail clips of newborn or two week old mice were subjected to genomic DNA extraction and genotyped by PCR (see Table S1 for primer sequences).

### Gene expression analysis in mouse embryos

*In situ* hybridization data were retrieved from the Eurexpress transcriptome atlas database [30]. Gene names *St14*, *Prss8*, and *F2rl1*, respectively, were individually searched to determine expression at embryonic day 14.5.

### Extraction of embryonic and perinatal tissues

Breeding females were checked for vaginal plugs in the morning and the day on which the plug was found was defined as the first day of pregnancy (E0.5). Pregnant females were euthanized in the mid-day at designated time points by CO_2_ asphyxiation. Embryos were extracted by Caesarian section and the individual embryos and placentas were dissected and processed. Only living embryos with detectable heartbeat were used for further analysis. Tail clips or yolk sacs of individual embryos were washed twice in phosphate buffered saline, subjected to genomic DNA extraction, and genotyped by PCR (Table S1). Newborn pups were euthanized by CO_2_ inhalation at 0°C. For histological analysis, the embryos and newborn pups were fixed for 24 h in 4% paraformaldehyde (PFA), processed into paraffin, sectioned, and stained with hematoxylin and eosin (H&E), or used for immunohistochemistry as described below. To evaluate the development of embryonic and placental tissues, H&E-stained midline sagittal embryonic and the midline cross sections from at least three *F2rl1^−/−^;St14^−/−^* and three corresponding littermate controls were inspected by a certified pathologist for possible abnormalities.

### Analysis of placental morphogenesis

Placental tissues from living E12.5 and E13.5 embryos were extracted and processed into paraffin as described above. To analyze the overall thickness of the placental labyrinth, a single midline cross-section at the level of the umbilical cord was stained with hematoxylin & eosin and the maximum perpendicular distance between the undifferentiated chorionic epithelium and the labyrinth-supporting spongiotrophoblast layer was measured under a light microscope by an investigator blinded to the genotypes of individual embryos. The volume of the placental labyrinth was estimated using Cavalieri's principle. Briefly, PFA-fixed placental tissue was embedded in paraffin, cross sections 200 µm apart covering the entire volume of the tissue were stained with h&e, scanned using ScanScope system (Aperio Technologies, Vista, CA), and the area of the labyrinth was measured on each section using Image J 1.46r software (National Institutes of Health, MD). The total volume of the labyrinth was then estimated as the sum of partial volumes calculated as area of the labyrinth on each section multiplied by 200 µm.

To evaluate branching of the fetal vasculature in the E12.5 and E13.5 placentas, a single midline cross section of each placenta at the level of the umbilical cord was immunostained with an anti-CD31 antibody (see below), followed by the manual counting of individual profiles of CD31-stained vessels within the placental labyrinth.

### Immunohistochemistry

Antigens from 5 µm paraffin sections were retrieved by incubation for 10 min at 100°C with 1 mM EDTA, pH 8.0 for CD31 staining, or by incubation for 20 min at 100°C in 0.01 M sodium citrate buffer, pH 6.0, for all other antigens. The sections were blocked with 2% bovine serum albumin (fraction V, MP Biomedicals, Solon, OH) in phosphate-buffered saline (PBS), and incubated overnight at 4°C with 2 ug/ml rabbit anti-human CD31 (Santa Cruz Biotechnology, Santa Cruz, CA), or sheep anti-human matriptase (R&D Systems, Minneapolis, MN), primary antibodies. Bound antibodies were visualized using biotin-conjugated anti-rabbit, or -sheep secondary antibodies (Vector Laboratories, Burlingame, CA) and a Vectastain ABC kit (Vector Laboratories) using 3,3′-diaminobenzidine as the substrate (Sigma-Aldrich, St. Louis, MO). All microscopic images were acquired on an Olympus BX40 microscope using an Olympus DP70 digital camera system (Olympus, Melville, NY).

### Protein extraction from mouse embryonic and newborn tissues

Placentas were extracted from embryos at E12.5 or E13.5. The embryonic portion of each placenta was manually separated from maternal decidua using a dissection microscope. The tissues were then homogenized in ice-cold 62.5 mM Tris/HCl, pH 6.8; 2% SDS; 10% glycerol buffer supplemented with 1× protease inhibitor cocktail (Sigma-Aldrich) and incubated on ice for 10 min. The lysates were centrifuged for 10 min at 20,000 g at 4°C to remove tissue debris and the supernatant was used for further analysis as described below.

### Western blotting

The protein concentration in cleared lysates from embryonic, placental, and newborn tissues was determined by BCA assay (Pierce, Rockford, IL). 80 µg of total protein was loaded on 4–12% reducing SDS-PAGE and analyzed by Western blotting using a polyclonal sheep anti-human matriptase (R&D Systems), mouse anti-cow desmoglein 1 and 2 (Fitzgerald Industries International, Acton, MA), mouse anti-human Gcm1 (Abcam, Cambridge, MA), rabbit anti-human syncytinA (SantaCruz Biotechnology), rabbit anti-human pan-cadherin, rabbit anti-human GAPDH (both Cell Signaling Technology, Danvers, MA), rabbit anti-human claudin-1, and mouse anti-human claudin-2 (both Invitrogen, Carlsbad, CA) primary antibodies, and goat anti-mouse, mouse anti-rabbit (both DakoCytomation), or donkey anti-sheep (Sigma-Aldrich) secondary antibodies conjugated to alkaline phosphatase. Alkaline phosphatase activity was then visualized using nitro-blue tetrazolium and 5-bromo-4-chloro-3′-indolyphosphate substrates. Where indicated, protein signal quantification was performed using ImageJ 1.46r software.

### β-galactosidase staining

Pregnant female mice from breeding pairs heterozygous β-galactosidase-tagged *F2rl1* knock-in mice were euthanized at E12.5 by cervical dislocation. Embryos were extracted by Caesarian section, and the individual embryos and placentas were dissected and placed in 2% PFA with 0.2% glutaraldehyde. Fixed tissues were stained overnight at room temperature in 1 mg/ml X-gal, 5 mM potassium ferricyanide, 5 mM potassium ferrocyanide, 2 mM magnesium chloride and 0.02% NP-40 in PBS. The tissues were post-fixed for 16 h in 4% PFA, embedded in paraffin, and sectioned. The sections were counterstained with nuclear fast red. All microscopic images were acquired on a Zeiss AxioImager Z1 light microscope (Carl Zeiss AG, Gottingen, Germany) using a Qicam FAST1394 digital camera from Qimaging.

### Placental transport and feto-maternal barrier assays

100 µl of PBS containing 1 uCi of 3-O-[methyl-^14^C]-D-glucose or [carboxyl-^14^C]-inulin (both Perkin-Elmer, Hanover, MD) was injected into the tail vein of pregnant female mice from *St14;F2rl1* double-heterozygous breeding pairs at 12.5 or 13.5 days of gestation. The mice were euthanized two min later by CO_2_ inhalation, the embryos were extracted as described above, weighted, and lysed overnight at 60°C in 500 µl of 2% potassium hydroxide solution. The lysate was then neutralized with 50 µl of 14 M hydrochloric acid, mixed with 10 ml of ScintiSafe scintillation liquid (Fisher Scientific, Pittsburg, PA), and [^14^C] activity was measured using Beckman LS6000IC scintillation counter (Beckman Coulter, Brea, CA). To evaluate the differences in placental function between the individual embryos, the activity was normalized to gram of embryonic tissue.

### Statistical analysis

The survival of newborn and pre-weaning mice from PAR-2/matriptase double heterozygous or PAR-2/prostasin double heterozygous breeding pairs was statistically evaluated by using chi-square analysis of the observed versus the expected distribution of mice wildtype, heterozygous, and deficient for PAR-2 (*F2rl1^+/+^*; *F2rl1^+/−^*; and *F2rl1^−/−^*, respectively) among living offspring carrying two (*St14^+/+^* or *Prss8^+/+^*), one (*St14^+/−^* or *Prss8^+/−^*), or no functional alleles (*St14^−/−^* or *Prss8^−/−^*) of the gene encoding the corresponding protease.

To evaluate the effect of matriptase or prostasin deficiency on the embryonic survival of PAR-2-deficient mice, chi-square analysis was performed on the observed versus the expected distribution of protease-expressing (*St14^+/+^* and *St14^+/−^*, or *Prss8^+/+^* and *Prss8^+/−^*, respectively) and protease-deficient (*St14^−/−^* or *Prss8^−/−^*) animals among the PAR-2-deficient (*F2rl1^−/−^*) embryos extracted at different embryonic stages, as indicated in the text.

Morphometric parameters of the placental development were analyzed using tissues from at least five control and five *F2rl1^−/−^;St14^−/−^* double-deficient animals and the observed values were statistically evaluated using a two-sample Student's t-test, two-tailed.

## Supporting Information

Figure S1 A combined loss of PAR-2 and matriptase leads to a decreased volume of placental labyrinth. (**A**). Schematic depiction of the structure of mid-gestational placenta (left) and the stereological technique used to estimate the total volume of the placental labyrinth bases on Cavalieri's principle (right). The mathematical formula used to calculate the labyrinth volume is shown on the right. am, allantoic mesenchyme; lb, labyrinth; tgc/sp, trophoblast giant cells and spongiotrophoblasts; dc, decidua. (**B**). Quantification of the labyrinth volume in PAR-2- and matriptase-expressing (control) and the double-deficient (*F2rl1^−/−^; St14^−/−^*) animals at E13.5. Loss if matriptase and PAR-2 expression led to 15% reduction in the volume (P = 0.06; Student t-test two-tailed, N = 3).
(TIF)

Figure S2 Expression of claudin-1 in PAR-2 and matriptase single-deficient placentas. Western blot analysis of claudin-1 expression in the placentas of the matriptase- and PAR-2-expressing (control, lanes 1 and 2), PAR-2-deficient (*F2rl1^−/−^*, lanes 3 and 4), and matriptase-deficient (*St14^−/−^*, lanes 5–7) mice at E13.5. The figure is a composite of samples run in parallel on two separate gels (indicated by white line). Expression of claudin-1 (arrowhead on the right-hand side) was not affected by a single loss of PAR-2 or matriptase activity. Positions of molecular weight markers (kDa) are shown on left.
(TIF)

Table S1 Sequences of PCR primers used for mouse genotyping.
(DOCX)

Table S2 Postnatal survival of *F2rl1^−/−^* mice in the offspring of *Spint2^+/−^;F2rl1^+/−^*×*Spint2^+/−^;F2rl1^+/−^;St14^+/−^* breeding pairs.
(DOCX)

Table S3 Prenatal survival of *F2rl1^−/−^* mice in the offspring of *F2rl1^+/−^;St14^+/−^*×*F2rl1^+/−^;St14^+/−^* breeding pairs.
(DOCX)

Table S4 Prenatal survival of *F2rl1^−/−^* mice in the offspring of *F2rl1^+/−^;Prss8^+/−^*×*F2rl1^+/−^;Prss8^+/−^* breeding pairs.
(DOCX)

Table S5 Embryonic survival of *F2rl1^−/−^;St14^−/−^* mice.
(DOCX)

Table S6 Embryonic survival of *F2rl1^−/−^;Prss8^−/−^* mice.
(DOCX)
