# Supplementary figures and images for: Regulation of Feto-Maternal Barrier by Matriptase- and PAR-2-Mediated Signaling Is Required for Placental Morphogenesis and Mouse Embryonic Survival
Source: PLoS Genet. 2014 Jul 31;10(7):e1004470. doi: 10.1371/journal.pgen.1004470 (PMC4117450; doi:10.1371/journal.pgen.1004470)

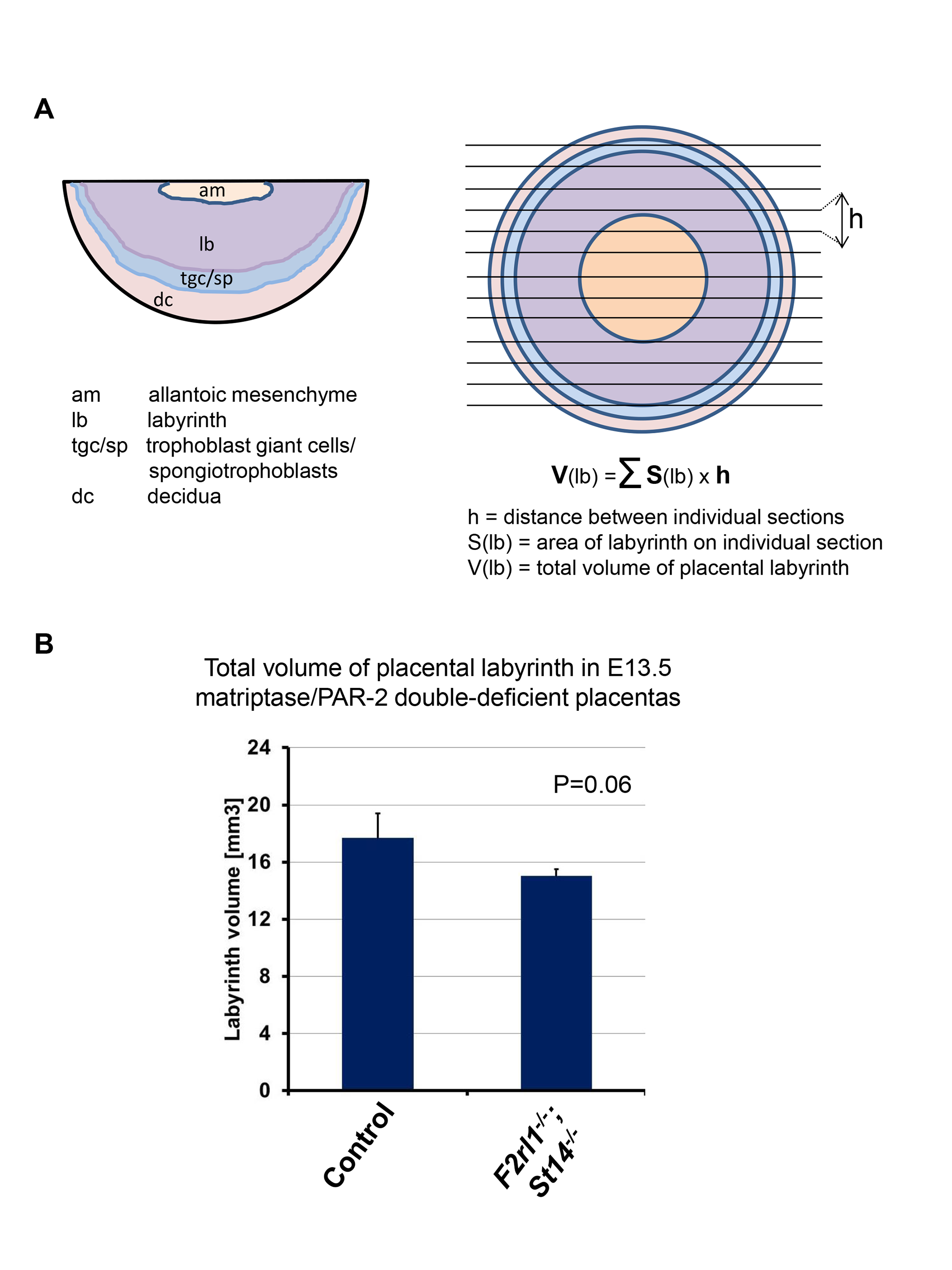

Supplement: Figure S1 — A combined loss of PAR-2 and matriptase leads to a decreased volume of placental labyrinth. (A). Schematic depiction of the structure of mid-gestational placenta (left) and the stereological technique used to estimate the total volume of the placental labyrinth bases on Cavalieri's principle (right). The mathematical formula used to calculate the labyrinth volume is shown on the right. am, allantoic mesenchyme; lb, labyrinth; tgc/sp, trophoblast giant cells and spongiotrophoblasts; dc, decidua. (B). Quantification of the labyrinth volume in PAR-2- and matriptase-expressing (control) and the double-deficient (F2rl1−/−; St14−/−) animals at E13.5. Loss if matriptase and PAR-2 expression led to 15% reduction in the volume (P = 0.06; Student t-test two-tailed, N = 3). (TIF) [file pgen.1004470.s001.tif]

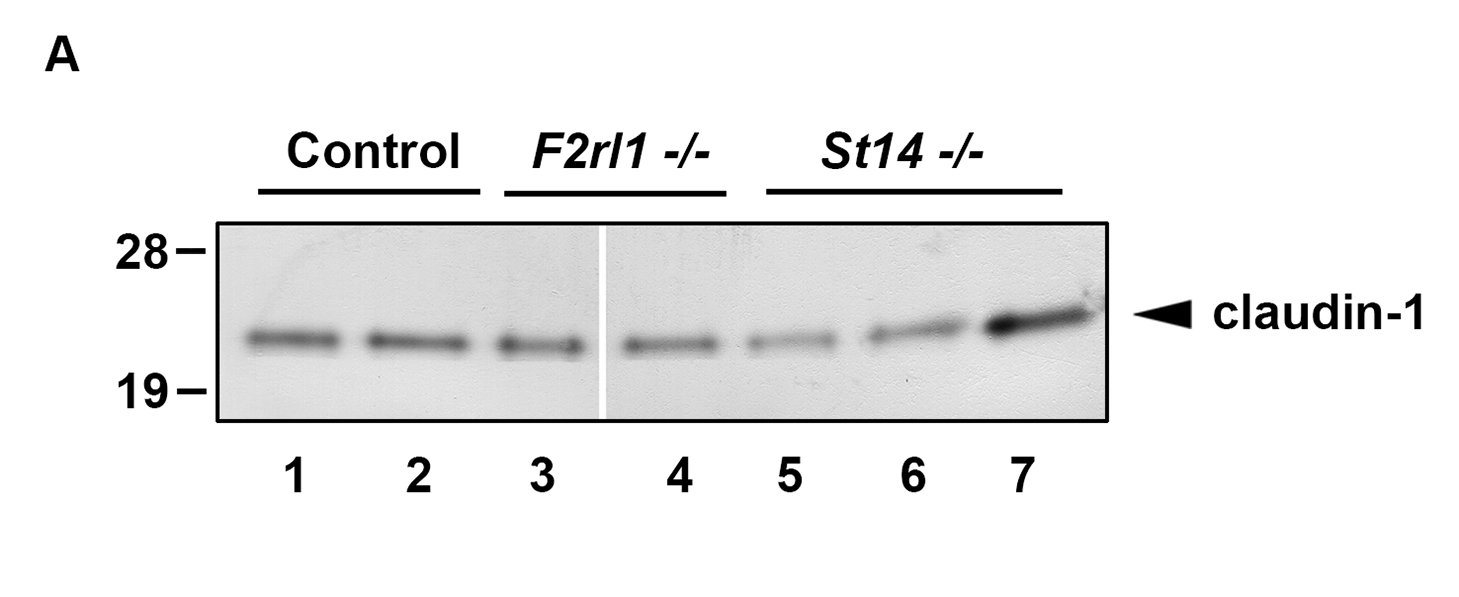

Supplement: Figure S2 — Expression of claudin-1 in PAR-2 and matriptase single-deficient placentas. Western blot analysis of claudin-1 expression in the placentas of the matriptase- and PAR-2-expressing (control, lanes 1 and 2), PAR-2-deficient (F2rl1−/−, lanes 3 and 4), and matriptase-deficient (St14−/−, lanes 5–7) mice at E13.5. The figure is a composite of samples run in parallel on two separate gels (indicated by white line). Expression of claudin-1 (arrowhead on the right-hand side) was not affected by a single loss of PAR-2 or matriptase activity. Positions of molecular weight markers (kDa) are shown on left. (TIF) [file pgen.1004470.s002.tif]
