# Supplementary material for: Regulation of Feto-Maternal Barrier by Matriptase- and PAR-2-Mediated Signaling Is Required for Placental Morphogenesis and Mouse Embryonic Survival
Source: PLoS Genet. 2014 Jul 31;10(7):e1004470. doi: 10.1371/journal.pgen.1004470 (PMC4117450; doi:10.1371/journal.pgen.1004470)
Supplement: Table S1 — Sequences of PCR primers used for mouse genotyping. (DOCX) [file pgen.1004470.s003.docx]

**Table S1.** Sequences of PCR primers used for mouse genotyping

| **Detection of *Spint2* alleles** | | | **Ta=55°C** |
| --- | --- | --- | --- |
| WT Forward | | 5’-aacacatttcaccaccatgc-3’ | |
| WT Reverse | | 5’-ccagactttcctaagtggg-3’ | |
| KO Forward | | 5’-atctgcaacctcaagctagc-3’ | |
| KO Reverse | | 5’-cagaaccagcaaactgaagg-3’ | |
|  |  | | |
| **Detection of *St14* alleles** | | | **Ta=55°C** |
| WT Forward | | 5’-cagtgctgttcagcttcctctt-3’ | |
| KO Forward | | 5’-gcatgctccagactgccttg-3’ | |
| WT and KO Reverse | | 5’-gtggaggtggagttctcatacg-3’ | |
|  |  | | |
| **Detection of *Prss8* alleles** | | | **Ta=55°C** |
| WT Forward | | 5’-ccctcacaatcctgacaatggc-3’ | |
| KO Forward | | 5’-ccaataaaccctcttgcagttgc-3’ | |
| WT and KO Reverse | | 5’-acttagccacactaagtgtccc-3’ | |
|  |  | | |
| **Detection of *F2rl1* alleles** | | | **Ta=55°C** |
| WT Forward | | 5’-ggtccaacagtaaggctgct-3’ | |
| KO Forward | | 5’-gccagaggccacttgtgtag-3’ | |
| WT and KO Reverse | | 5’-tcaaagactgctggtggttg-3’ | |
|  |  | | |
| **Detection of *Meox2-Cre* allele** | | | **Ta=55°C** |
| Cre Forward | | 5’-ggacatgttcagggatcgccaggcg-3’ | |
| Cre Reverse | | 5’-gcataaccagtgaaacagcattgctg-3’ | |
