# Supplementary material for: Regulation of Feto-Maternal Barrier by Matriptase- and PAR-2-Mediated Signaling Is Required for Placental Morphogenesis and Mouse Embryonic Survival
Source: PLoS Genet. 2014 Jul 31;10(7):e1004470. doi: 10.1371/journal.pgen.1004470 (PMC4117450; doi:10.1371/journal.pgen.1004470)
Supplement: Table S2 — Postnatal survival of F2rl1−/− mice in the offspring of Spint2+/−;F2rl1+/−×Spint2+/−;F2rl1+/−;St14+/− breeding pairs. (DOCX) [file pgen.1004470.s004.docx]

**Table S2.** Postnatal survival of *F2rl1^-/-^* mice in the offspring of *Spint2^+/-^;F2rl1^+/-^* x *Spint2^+/-^;F2rl1^+/-^;St14^+/-^* breeding pairs.

|  | **Number of living animals at weaning**  **Observed (Expected^1^)** | | | **Relative survival of *F2rl1^-/-^* animals** | **P value**  **(chi-square)^2^** |
| --- | --- | --- | --- | --- | --- |
|  | ***F2rl1^+/+^*** | ***F2rl1^+/-^*** | ***F2rl1^-/-^*** | **(% of expected)** |  |
| ***St14^+/+^***  ***St14^+/-^*** | 91 (90.75)  91 (85.75) | 190 (181.5)  218 (171.5) | 82 (90.75)  34 (85.75) | 90  40 | 0.53  <0.0001 |

^1^ Mendelian distribution based on parental genotypes (*F2rl1^+/-^* x *F2rl1^+/-^;St14^+/-^* breeding pairs)

^2^ Observed vs. expected distribution of animals wildtype, heterozygous, and deficient for PAR-2 (*F2rl1^+/+^*; *F2rl1^+/-^*; and *F2rl1^-/-^*, respectively) among the living weaning-age offspring carrying two (*St14^+/+^*), or one (*St14^+/-^*) functional allele of matriptase.
