# Supplementary material for: Regulation of Feto-Maternal Barrier by Matriptase- and PAR-2-Mediated Signaling Is Required for Placental Morphogenesis and Mouse Embryonic Survival
Source: PLoS Genet. 2014 Jul 31;10(7):e1004470. doi: 10.1371/journal.pgen.1004470 (PMC4117450; doi:10.1371/journal.pgen.1004470)
Supplement: Table S3 — Prenatal survival of F2rl1−/− mice in the offspring of F2rl1+/−;St14+/−×F2rl1+/−;St14+/− breeding pairs. (DOCX) [file pgen.1004470.s005.docx]

**Table S3.** Prenatal survival of *F2rl1^-/-^* mice in the offspring of *F2rl1^+/-^;St14^+/-^* x *F2rl1^+/-^;St14^+/-^* breeding pairs.

|  | **Number of living animals at birth**  **Observed (Expected^1^)** | | | **Relative survival of *F2rl1^-/-^* animals** | **P value**  **(chi-square)^2^** |
| --- | --- | --- | --- | --- | --- |
|  | ***F2rl1^+/+^*** | ***F2rl1^+/-^*** | ***F2rl1^-/-^*** | **(% of expected)** |  |
| ***St14^+/+^***  ***St14^+/-^***  ***St14^-/-^*** | 23 (21.25)  40 (39.75)  14 (7) | 43 (42.5)  103 (79.5)  14 (14) | 19 (21.25)  16 (39.75)  0 (7) | 90  40  0 | 0.82  <0.001  <0.001 |

^1^ Mendelian distribution based on parental genotypes (*F2rl1^+/-^;St14^+/-^* x *F2rl1^+/-^;St14^+/-^* breeding pairs)

^2^ Observed vs. expected distribution of animals wildtype, heterozygous, and deficient for PAR-2 (*F2rl1^+/+^*; *F2rl1^+/-^*; and *F2rl1^-/-^*, respectively) among the living weaning-age offspring carrying two (*St14^+/+^*), one (*St14^+/-^*), or no (*St14^-/-^*) functional allele of matriptase.
