# Supplementary material for: Regulation of Feto-Maternal Barrier by Matriptase- and PAR-2-Mediated Signaling Is Required for Placental Morphogenesis and Mouse Embryonic Survival
Source: PLoS Genet. 2014 Jul 31;10(7):e1004470. doi: 10.1371/journal.pgen.1004470 (PMC4117450; doi:10.1371/journal.pgen.1004470)
Supplement: Table S5 — Embryonic survival of F2rl1−/−;St14−/− mice. (DOCX) [file pgen.1004470.s007.docx]

**Table S5.** Embryonic survival of *F2rl1^-/-^; St14^-/-^* mice.

| **Age** | **Survival of *F2rl1^-/-^* embryos**  **Observed (Expected^1^)** | | | **Relative survival of *F2rl1^-/-^*; *St14^-/-^* embryos** | **P value**  **(chi-square)^2^** |
| --- | --- | --- | --- | --- | --- |
|  | ***St14^+/+^*** | ***St14^+/-^*** | ***St14^-/-^*** | **(% of expected)** |  |
| **E10.5**  **E11.5**  **E12.5**  **E13.5**  **E14.5**  **E15.5** | 4 (5.25)  5 (5.25)  10 (11.25)  23 (18.5)  7 (7.25)  9 (5.5) | 12 (10.5)  10 (10.5)  24 (22.5)  40 (37)  21 (14.5)  13 (11) | 5 (5.25)  6 (5.25)  11 (11.25)  11 (18.5)  1 (7.25)  0 (5.5) | 95  114  98  59  14  0 | 0.90  0.71  0.93  0.04  0.007  0.007 |

^1^ Mendelian distribution based on parental genotypes (*F2rl1^+/-^;St14^+/-^* x *F2rl1^+/-^;St14^+/-^* and *F2rl1^-/-^;St14^+/-^* x *F2rl1^+/-^;St14^+/-^* breeding pairs)

^2^ Observed vs. expected distribution of matriptase-deficient (*St14^-/-^*) and matriptase-expressing (*St14^+/+^* and *St14^+/-^*) mice among *F2rl1*-deficient embryos
