# Supplementary material for: Regulation of Feto-Maternal Barrier by Matriptase- and PAR-2-Mediated Signaling Is Required for Placental Morphogenesis and Mouse Embryonic Survival
Source: PLoS Genet. 2014 Jul 31;10(7):e1004470. doi: 10.1371/journal.pgen.1004470 (PMC4117450; doi:10.1371/journal.pgen.1004470)
Supplement: Table S6 — Embryonic survival of F2rl1−/−;Prss8−/− mice. (DOCX) [file pgen.1004470.s008.docx]

**Table S6.** Embryonic survival of *F2rl1^-/-^;Prss8^-/-^* mice.

| **Age** | **Survival of *F2rl1^-/-^* embryos**  **Observed (Expected^1^)** | | | **Relative survival of *F2rl1^-/-^; Prss8^-/-^* embryos** | **P value**  **(chi-square)^2^** |
| --- | --- | --- | --- | --- | --- |
|  | ***Prss8^+/+^*** | ***Prss8^+/-^*** | ***Prss8^-/-^*** | **(% of expected)** |  |
| E11.5  E12.5  E13.5  E14.5  E15.5 | 5 (5.25)  10 (6.25)  9 (12.75)  9 (5.75)  12 (5.75) | 9 (10.5)  10 (12.5)  36 (25.5)  13 (11.5)  11 (11.5) | 7 (5.25)  5 (6.25)  6 (12.75)  1 (5.75)  0 (5.75) | 133  80  47  17  0 | 0.38  0.56  0.03  0.03  0.006 |

^1^ Mendelian distribution based on parental genotypes (*F2rl1^+/-^;Prss8^+/-^* x *F2rl1^+/-^;Prss8^+/-^* and *F2rl1^-/-^;Prss8^+/-^* x *F2rl1^+/-^;Prss8^+/-^* breeding pairs)

^2^ Observed vs. expected distribution of prostasin-expressing (*Prss8^+/+^* and *Prss8^+/-^*) and prostasin-deficient (*Prss8^-/-^*) mice among *F2rl1*-deficient embryos
